# The extracellular serine protease from *Staphylococcus epidermidis* elicits a type 2-biased immune response in atopic dermatitis patients

**DOI:** 10.3389/fimmu.2024.1352704

**Published:** 2024-06-04

**Authors:** Goran Abdurrahman, Rebecca Pospich, Leif Steil, Manuela Gesell Salazar, Juan José Izquierdo González, Nicole Normann, Daniel Mrochen, Christian Scharf, Uwe Völker, Thomas Werfel, Barbara M. Bröker, Lennart M. Roesner, Lidia Gómez-Gascón

**Affiliations:** ^1^ Institute of Immunology, University Medicine Greifswald, Greifswald, Germany; ^2^ Department of Dermatology and Allergy, Hannover Medical School, Hannover, Germany; ^3^ Department of Functional Genomics, University Medicine Greifswald, Greifswald, Germany; ^4^ Department of Otorhinolaryngology, Head and Neck Surgery, University Medicine Greifswald, Greifswald, Germany; ^5^ Cluster of Excellence RESIST (EXC 2155), Hannover Medical School, Hannover, Germany

**Keywords:** *Staphylococcus epidermidis*, atopic dermatitis, allergy, IgE, Th2 cells, protease, Esp

## Abstract

**Background:**

Atopic dermatitis (AD) is a chronic, relapsing inflammatory skin disease with skin barrier defects and a misdirected type 2 immune response against harmless antigens. The skin microbiome in AD is characterized by a reduction in microbial diversity with a dominance of staphylococci, including *Staphylococcus epidermidis* (*S. epidermidis*).

**Objective:**

To assess whether *S. epidermidis* antigens play a role in AD, we screened for candidate allergens and studied the T cell and humoral immune response against the extracellular serine protease (Esp).

**Methods:**

To identify candidate allergens, we analyzed the binding of human serum IgG4, as a surrogate of IgE, to *S. epidermidis* extracellular proteins using 2-dimensional immunoblotting and mass spectrometry. We then measured serum IgE and IgG1 binding to recombinant Esp by ELISA in healthy and AD individuals. We also stimulated T cells from AD patients and control subjects with Esp and measured the secreted cytokines. Finally, we analyzed the proteolytic activity of Esp against IL-33 and determined the cleavage sites by mass spectrometry.

**Results:**

We identified Esp as the dominant candidate allergen of *S. epidermidis*. Esp-specific IgE was present in human serum; AD patients had higher concentrations than controls. T cells reacting to Esp were detectable in both AD patients and healthy controls. The T cell response in healthy adults was characterized by IL-17, IL-22, IFN-γ, and IL-10, whereas the AD patients’ T cells lacked IL-17 production and released only low amounts of IL-22, IFN-γ, and IL-10. In contrast, Th2 cytokine release was higher in T cells from AD patients than from healthy controls. Mature Esp cleaved and activated the alarmin IL-33.

**Conclusion:**

The extracellular serine protease Esp of *S. epidermidis* can activate IL-33. As an antigen, Esp elicits a type 2-biased antibody and T cell response in AD patients. This suggests that *S. epidermidis* can aggravate AD through the allergenic properties of Esp.

## Graphical Abstract

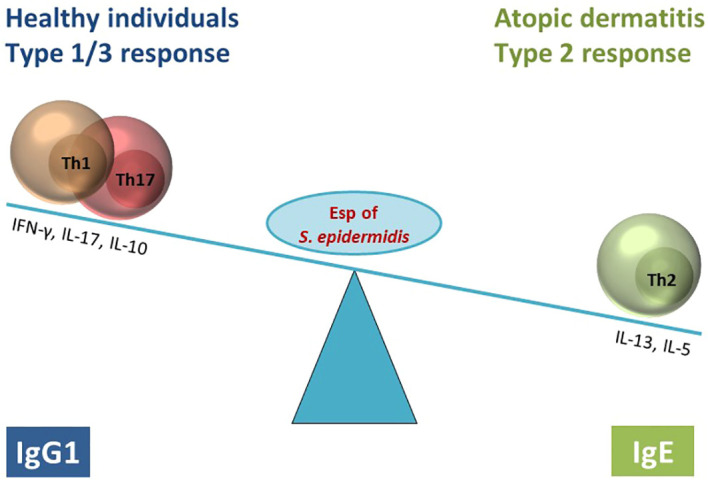

## 1 Introduction

Atopic dermatitis (AD) is a chronic, relapsing inflammatory skin disease affecting up to 20% of children and 5-10% of adults worldwide (1–3). Disease course and disease severity are highly variable in AD and the pathogenesis is not fully resolved. According to an integrated concept, three main factors contribute to pathogenesis: skin barrier defects, inflammation and dysbiosis. These are commonly observed in AD in lesional and to a lesser extent in nonlesional skin, and they may reinforce one another (1, 3–5). Barrier defects, for example, facilitate the penetration of allergens and other environmental stressors into the skin, promoting sensitization and inflammation, which further weakens the skin barrier (5, 6). The inflammation in AD is type 2-biased, characterized by IgE production and increased numbers of Th2 cells that release IL-4, IL-5, and IL-13 (7, 8).

IL-33 belongs to the family of IL-1 cytokines and acts as an alarmin, i.e., damage-associated molecular pattern (DAMP), because it is released during cell death or in response to various environmental stressors (9–12). In AD increased amounts of IL-33 are found in the skin; the main producers are keratinocytes (13). The cytokine acts via the cellular receptor ST2 and has a strong immune polarizing effect, inducing the production of type 2 cytokines in innate and adaptive immune cells. Thus, IL-33 contributes to the priming and effector phases of allergic inflammation (10, 14, 15). The biological activity of IL-33 is regulated by proteases of the host and the environment. IL-33 has no signal peptide and is released in its full-length form, IL-33_1–270_, which is biologically active. N-terminal truncation through cleavage at the central domain generates variants of IL-33 with up to 30-fold increased biological activity (10). Notably, many allergens have protease activity and can cleave full-length human IL-33_1–270_ to generate mature forms: IL-33_95–270_, IL-33_99–270_, and IL-33_109–270_ (11). Proteases of neutrophils and mast cells also activate IL-33 (11, 16, 17). Cleavage by caspases, in contrast, inactivates the alarmin (18, 19).

Another feature of AD is dysbiosis with an increased abundance of staphylococci in the skin microbiome, mainly *Staphylococcus aureus* (*S. aureus*) but also *Staphylococcus epidermidis* (*S. epidermidis*) (3, 20, 21). The role of *S. epidermidis* is complex (22, 23). The bacterium has long been considered beneficial in AD, mainly due to its ability to counteract *S. aureus* (*S. aureus*) and degrade its biofilms (24–28). On the other hand, *S. epidermidis* has been found at increased density on lesional skin in AD, often but not always together with *S. aureus* (20, 29–32). Moreover, *S. epidermidis* strains that express the protease EcpA can cause severe skin lesions in a mouse model of AD, indicating that overabundance of *S. epidermidis* can be as harmful as *S. aureus* (31).

Bacteria have a dual role in allergy. Typically, they are associated with protection, but colonization and infection with certain bacterial species increase the risk of allergic diseases (33). Therefore, bacterial antigens are being examined for their allergenic potential. As an example, serine protease-like proteins (Spls) from *S. aureus* elicit an allergic immune response in asthma and cystic fibrosis. Many patients have specific Th2 cells as well as Spl-binding serum IgE (34, 35). In mice, intra-tracheal administration of SplD induced eosinophilic lung inflammation, which was dependent on IL-33 (34, 36). It is not known whether *S. epidermidis* can also produce allergenic proteins.

The extracellular serine protease (Esp) is encoded in the genome of 96% of clinical *S. epidermidis* isolates, but is not found in other staphylococci (28, 37). The enzyme is released by the bacteria in an inactive form, pro-Esp, also known as glutamyl endopeptidase of *S. epidermidis* (GluSE), that is processed into the mature Esp by unknown proteases that remove the N-terminal pro-peptide of 66 amino acids (38, 39). Esp has been implicated in bacterial interference. The protease destroys *S. aureus* biofilms (26) and selectively degrades several human receptor proteins involved in *S. aureus* colonization or infection (24, 26). Moreover, the protease degrades the complement component C5 and human keratin (40).

We hypothesized that *S. epidermidis* may worsen AD through the release of allergenic proteins. Hence, we studied the immune response against *S. epidermidis* in AD patients and healthy volunteers and characterized the humoral and T cell response against its serine protease Esp in detail.

## 2 Materials and methods

### 2.1 Human subjects

The study was approved by the responsible Ethics Committees of Hannover Medical School and University Medicine Greifswald (IIIUV 23/06a, BB007/17, MHH7565). All human study participants gave their written informed consent. Serum samples were obtained from 50 atopic dermatitis (AD) patients at the Department of Dermatology and Allergy, Hannover Medical School, Germany. The median age of AD patients was 31 y; 24 patients (48%) were male, 26 (52%) were female (**Supplementary Table S1**). Thirty serum samples from healthy volunteers were collected in-house at the Department of Immunology, University Medicine Greifswald, Germany. The healthy subjects’ median age was 23 y; 13 (28.2%) were male and 33 (71.8%) female. The samples were kept at -80°C until analysis and were used to determine antibody binding (IgG1, IgG4, and IgE) to the target proteins of *S. epidermidis*.

For T cell stimulation assays, EDTA blood samples were taken from 8 AD patients (**Supplementary Table S2**) and 16 healthy individuals. The AD blood samples were obtained from the Department of Dermatology and Allergy, Hannover Medical School, Germany. At the same time, the healthy subjects were recruited in both Hannover and Greifswald, Germany. The median age of AD patients was 27 y; 5 patients (62.5%) were male, and 3 (37.5%) were female. The median age of healthy T cell donors was 30.5 y; 7 individuals (43.75%) were male and 9 (56.25%) were female.

### 2.2 Bacterial strains and bacterial extracellular proteins

The *S. epidermidis* reference strain RP62A was used in this study. The bacteria were plated on a Columbia agar blood base containing 5% (v/v) sheep blood and grown at 37°C. One single colony was grown in 10 mL of Tryptic Soy Broth (TSB) at 37°C and 200 rpm overnight and then inoculated into sterile TSB medium to an OD_595_ of 0.05. The culture was incubated at 37°C until 3 hours after the stationary phase was reached.

Extracellular proteins (ECPs) of *S. epidermidis* were prepared as previously described (41). Briefly, the bacterial culture supernatants were collected by centrifugation at 8000 g for 10 min at 4°C and filtered through a 0.22-μm membrane to remove residual bacteria. The supernatants containing the ECPs were precipitated using trichloroacetic acid solution (100% TCA). The filtrates were mixed 9:1 (v/v) with pre-chilled 100% TCA solution and incubated on ice overnight at 4°C. After centrifugation at 8000 g for 1 hour at 4°C, the pellets were resuspended in 70% ethanol and washed four times and at the end washed once in 96% ethanol. The resulting pellets were air-dried. Finally, the precipitated proteins were dissolved in rehydration buffer (8M urea, 2M thiourea, 2% CHAPS). The protein concentration was determined by the Bradford method, and the extracts were stored at -80°C until further analysis.

### 2.3 Western blotting

One-dimensional (1D) immunoblotting was performed with an automated capillary-based blotting system (PeggySue Simple Western Assay). ECPs of *S. epidermidis* strain RP62A were used at a concentration of 1 mg/mL. Human serum (1:50) and horseradish peroxidase (HRP)-conjugated anti-human IgG4 secondary antibody (Thermofisher, A-10654) at a concentration of 10 μg/mL were diluted in the provided blocking diluent (ProteinSimple). The resulting signals were analyzed with Compass software 2.6.5 (ProteinSimple).

Two-dimensional (2D) immunoblotting was performed to visualize the binding of serum IgG4 to the ECPs of *S. epidermidis*. The procedure was performed as previously described in (42, 43), with the following adaptations. ECPs were treated with the 2D Clean-up kit (GE Healthcare) following the manufacturer’s instructions. Proteins were separated by 2D electrophoresis. In the first dimension, proteins were separated according to their isoelectric point using 11-cm Immobiline DryStrips (IPG, Immobilized pH Gradient, pH range 4-7 (Cat no. 18-1016-60); and 6-11 (Cat no. 17-6001-95; GE Healthcare). IPG strips were rehydrated for 16 hours in rehydration sample buffer (7M urea, 2M thiourea, 4% CHAPS, 20mM DTT, 1.6% ampholytes) containing 160 μg of the protein samples in a total volume of 200 μL. Isoelectric focusing was performed using a Bio-Rad PROTEAN IEF cell (Bio-Rad). The focusing was conducted at 20°C by a stepwise increase of the voltage as follows: 300 V for 0.5 h, 3500 V for 2 hours, and increases in steps of 3500 V until 15,000 V or 12,000 V was finally reached, for 4-7 IPG strips or 6-11 IPG strips, respectively. Then each IPG strip was incubated in 5 mL of equilibration buffer 1 (50mM Tris−HCl pH 8.8; 6M urea, 2%SDS, 20% v/v glycerol, 2% DTT) for 15 min with shaking and then in 5 mL of equilibration buffer 2 (50mM Tris−HCl pH 8.8; 6M urea, 2% SDS, 20% v/v glycerol, 135mM iodoacetamide) for another 15 minutes with shaking.

In the second dimension, proteins were resolved using SDS-PAGE (12,5% polyacrylamide gel) according to molecular mass, and 0.5 W per gel was applied overnight until the tracking dye reached the gel bottom.

The separated proteins were transferred onto a polyvinylidene difluoride (PVDF) membrane (Merck Millipore, IPVH00005). Electrotransfer time was 1 hour and 40 minutes at 80 mA per gel. After blocking with 5% skim milk powder in Tris-buffered saline/Tween buffer (20 mmol/L Tris-HCl, 137 mmol/L NaCl, and 0.1% [vol/vol] Tween 20 [pH 7.6]) for 1 hour at room temperature, membranes were incubated with pooled AD serum, diluted 1:500 in blocking buffer. IgG4 binding was visualized by incubation with HRP-conjugated mouse anti-human IgG4 (Thermofisher, A-10654, 0.1 μg/mL; 1 h at room temperature). Membranes were developed using the SuperSignal™ West Femto Maximum Sensitivity Substrate (Thermo Scientific™, 34095) and then visualized with the high sensitivity Chemiluminescence Imager Octoplus QPLEX (Dyeagnostics, Germany).

Western blotting was also performed to confirm the mature Esp generation and to visualize the IL-33 cleavage pattern (see below).

Briefly, for Esp detection, 0.5 μg of proEsp or 0.5 μg of mature Esp were run on 12 SDS-PAGE gels. To visualize IL-33 cleavage-patterns, the required amounts Esp and IL-33 were loaded onto a 12% SDS-PAGE gel. The proteins were then blotted onto PVDF membranes. Next, the membranes were incubated with blocking buffer Roti-Block (Roth, A151.1) for one hour at room temperature. This blocking buffer is devoid of protease activity. The blots were then incubated with primary antibodies diluted in Roti-Block blocking buffer (for Esp detection: mouse anti-His antibody, Biolegend 652501, 1:2000 dilution; for IL-33 detection: mouse anti-human IL-33 monoclonal antibody, Cayman chemical company 10809, 1:4000 dilution). The blots were incubated overnight at 4°C, with slow shaking. On the next day, the membranes were washed three times with wash buffer [TBS-Tween20 (0.05% Tween20)] and incubated with the HRP-conjugated goat anti-mouse IgG (South Biotech, 1030-05) at 1:50,000 dilution in Roti-block buffer for 1 hour at room temperature. After three washing steps, the blots were incubated with SuperSignalTM West Femto maximum sensitivity substrate (Thermo Scientific, 34095) for 5 minutes, and the chemiluminescence signals were acquired with a high sensitivity Chemiluminescence Imager Octoplus QPLEX (Dyeagnostics).

### 2.4 Mass spectrometry

#### 2.4.1 Identification of proteins of interest

Protein spots of interest were manually excised from the 2D PVDF membranes and manually digested. Briefly, membrane pieces were destained by one wash at room temperature for 30 min with 50% methanol. Then, the pieces were trypsin-digested: 10 μL of 20 mM ammonium bicarbonate (ABC buffer) in 30% (v/v) acetonitrile (ACN) were added to the membrane pieces together with 4 µL trypsin solution (10 ng/μL) and left at 37°C overnight. The supernatants were collected, and 20 µL of 80% ACN was added to the membrane pieces and incubated for 30 min at room temperature. The supernatant was collected again and both samples were pooled. The samples were dried entirely by spinning in a SpeedVac Vacuum Concentrator, and 12 µL buffer A (0.1% acetic acid, 2% ACN) was added to the micro-vials, which were stored at -20°C until MS analysis.

Chromatographic separation of the peptides was performed on a reverse-phase nano-Acquity UPLC column (1.7 μm, 100 μm i.d. × 100 mm, Waters GmbH, Eschborn, Germany) using a 25 min gradient ranging from 5 to 70% ACN in 0.1% aqueous acetic acid solution at a flow rate of 400 nL/min. The nano-LC column was linked by electrospray ionization to a LTQ-Orbitrap Velos mass spectrometer (Thermo Scientific, San Jose, IL, U.S.A.). Precursor ions of m/z range 325–1525 (r=30.000) were subjected to data-dependent MS/MS fragmentation of the 20 most intense precursor peaks in the ion trap at collision-induced energy (CID) of 35%. Repetitive MS/MS acquisition was eliminated by setting dynamic exclusion of 60 s for already selected precursors (**Supplementary Table S3**).

Mascot search engine (version 2.6.2, Matrix Science) was used via the Proteome Discoverer (version 2.2, Thermo Scientific) to identify the proteins of interest. *S. epidermidis* specific databases were obtained from Uniprot (https://www.uniprot.org) and NCBI (https://www.ncbi.nlm.nih.gov).

#### 2.4.2 N-terminal enrichment analysis for cleavage site determination

Sample labeling and digestion were performed as described previously (44, 45) with slight modifications.

After incubation of 10 µg IL-33 with or without 10 µg Esp, both provided in PBS, at 37°C for 30 min, the reaction was stopped by reducing the sample for 30 min in the dark at 37°C with 10 mM DTT (100 mM DTT in 200 mM HEPES pH 7.0). After that, the samples were alkylated with 50 mM IAA (500 mM IAA in 200 mM HEPES pH 7.0) for 20 min in the dark at room temperature.

The samples were bound to a SP3 bead mixture (protein/bead ratio 1:10) at a final ethanol concentration of 80% (v/v) by incubation at room temperature in a thermoshaker (Eppendorf AG) at 1500 rpm for 18 min. The beads were separated from the supernatant by incubation on a magnetic stand for 2 min and washed twice by resuspension in 90% (v/v) ethanol and binding to the magnetic stand in between. Finally, the remaining ethanol was removed, and the beads were resuspended in 200 mM HEPES pH 7.0.

All samples were dimethyl labelled using light (CH_2_O) and heavy (^13^CD_2_O) formaldehyde and NaBH_3_CN (Sigma Aldrich, 252549, 596388, 156159). Samples were incubated in 40 mM formaldehyde and 20 mM NaBH_3_CN for 1 h at 37°C once, then fresh formaldehyde and NaBH_3_CN was added, and the incubation was prolonged for 1 h at 37°C. The reaction was stopped by adding 2 M Tris pH 6.8 to a final concentration of 600 mM and a 3 h incubation at 37°C. All incubations were performed in a thermoshaker (Eppendorf AG) at 700 rpm after adding components to the mixture and spinning down the samples; the beads were solubilized by placing the tube into a water sonification bath (Bandelin) for 30 s. Finally, the light labelled IL-33 samples were combined with the heavy labelled IL-33 + Esp samples before digestion.

Before digestion, the combined samples were bound to fresh SP3 beads (protein/bead ratio 1:5) as described above. The digestion was performed by adding chymotrypsin (25 ng/µL in 200 mM HEPES pH 8.0, ratio 1:200) to the bead bound protein samples and incubation for 4 h at 37°C. The peptides were separated from the beads by spinning the beads down 14000 g at room temperature and attaching them to the magnetic device. To obtain the whole supernatant, this process was done twice. Before Undecanal labelling of the peptide mixture, 5% of the sample was retained as pre-control and prepared for MS-measurement by adding 5% acetic to a final concentration of 1%.

In order to tag the free N-termini created by the chymotrypsin cleavage, the peptide mixture was labelled with undecanal (Sigma Aldrich, U2202) using NaBH3CN. Therefore, 100% ethanol to a final concentration of 40% (v/v) and undecanal in a protein/undecanal ratio (w/w) 1:30 was added to the mixture. The pH was checked and adjusted to pH 7-8 by adding 0.1% TFA in 40% ethanol. The reaction was incubated at 37°C in a thermomixer (Eppendorf AG) at 800 rpm for 1 h.

The removal of the undecanal labelled peptides was archived by using Sep-Pak C18 Cartridges (Waters). First, the cartridges were equilibrated rinsing with 1 mL of 100% methanol followed by 1 mL 0.1% TFA in 40% ethanol, then the sample was applied to the cartridge and rinsed with 200 µL 0.1% TFA in 40% ethanol. Both of the flow-throughs were combined and freeze-dried by lyophilization. After lyophilization, the samples were resuspended in 1% acetic acid.

Before measuring the samples by mass spectrometry, they were desalted using ZipTip-µC18 tips (Millipore), concentrated by evaporation under vacuum and subsequently resolved in 0.1% acetic acid, 2% acetonitrile (ACN).

Chromatographic separation and mass spectrometry were performed as described above, using a different gradient, provided in **Supplementary Table S3**.

#### 2.4.3 Data analysis

Proteome discoverer software (version 2.4, Thermo Scientific) was used to analyze the raw data. Ratios of the obtained peptide intensities were calculated, and peptide ratios between the three Esp cleaved samples and the three control IL-33 samples having an adjusted p-value > 0.05 were considered as significant (see **Supplementary Table S4**).

### 2.5 Recombinant proteins and enzyme assays

Recombinant Esp and GehD were cloned from genomic DNA of *S. epidermidis* strain RP62A using Gibson assembly protocols. The proteins were expressed in *E. coli* BL21 as recombinant proteins with C-terminal His-tag. The following primers were used to amplify the DNA sequence of Esp (Forward: ATGGTAGGTCTCAAATGAAAACCGATACAGAAAGCCATAATC, Reverse: ATGGTAGGTCTCAGCGCTCTGAATATTTATATCAGGTATATTGTTT) and GehD (Forward: gaaggagatatacaaatgGCTGAAATGACACAATCATC, Reverse: cgatcctctagcgctCTTACGTGTAATACCATCTAAC) with overhanging sequences compatible with the MCS region of the pASK-IBA33plus vector (IBA Lifesciences).

In the case of Esp, the vector pASK-IBA33plus was cut with the restriction enzyme BsaI (NEB, R3733) at 50°C for 1 hour in NEBuffer, and the reaction was stopped at 65°C for 20 min. In the case of GehD, the vector was linearized by PCR using linearization primers provided by the manufacturer. Later, the products’ ligation was performed using the Fast-Link™ DNA Ligation Kit (Epicenter, LK0750H) according to the manufacturer’s instructions. 200 μL of ligation product was transferred into chemically competent DH5α E. coli cells using standard molecular biology protocols. Recombinant colonies were randomly selected and checked for the presence of the inserted sequences by PCR. Next, pASK-IBA33plus-Esp and pASK-IBA33plus-GehD were transfected into chemical competent *E.coli* BL21 using standard protocols.

Protein expression was induced with anhydrotetracycline (AHT, diluted 1:10000; BA Lifesciences, 2-0401-001). The cells were harvested by centrifugation and lysed by sonication. The mixture was filtered and the protein extract was loaded on HisTrap nickel chromatography columns (HisTrap™ High Performance; GE Healthcare, 17-5248-02) using the ÄKTA Protein Purification System (Cytiva) according to the manufacturer’s instructions. The proteins were eluted with elution buffer (20 mM sodium phosphate, 0,5M sodium chloride, 500 mM imidazole, pH 7.4) using an isocratic elution program. The protein preparations were more than 95% pure, and the identity of the resulting protein was confirmed by MALDI-TOF MS analysis. For the T cell stimulation assays, LPS was rigorously depleted from the Esp preparations using an LPS depletion kit (Hyglos, Germany 800008). The final LPS concentration in the assays was < 0.1 EU/ml.

#### 2.5.1 Generation of mature Esp

The recombinant Esp was cloned and expressed in its inactive form, pro-Esp. For the protease to be activated, its 66-residue long propeptide has to be removed. Pro-Esp was processed into mature Esp by treating it with commercial thermolysin as previously described (26, 39). Briefly, pro-Esp (1 mg/mL) was incubated with thermolysin (Sigma, T7902-25G) (100 μg/mL) at 37°C for 3 hours to remove the N-terminal 66 amino acid long propeptide. The mature Esp was purified by affinity chromatography through its intact C-terminal His-tag. The identity of the products was confirmed by SDS-PAGE and Western blotting. In order to block residual thermolysin that might have survived the purification step, a final concentration of 5 mM of EDTA was added to the mature Esp preparations.

#### 2.5.2 Casein cleavage assay

The proteolytic activity of the activated Esp was confirmed in a casein cleavage assay. Briefly, casein from bovine milk (Sigma, C3400-500G) was prepared in 50 mM HEPES buffer (Sigma, H3375-250G) at a 1 mg/mL concentration. Then 1 μg of casein was mixed with 1 μg mature Esp in 20 μL PBS in the presences of 5 mM EDTA for 30 minutes at 37°C with slow shaking. The mixture was separated by 12% SDS-PAGE. The gel was stained with FlamingoTM Fluorescent Gel Stain (Bio-Rad,64217198) according to the manufacturer’s instructions. The fluorescent signal was acquired with a G200 filter of Imager Octoplus QPLEX (Dyeagnostics, Germany).

#### 2.5.3 IL-33 cleavage assay

For the IL-33 cleavage assay, full-length recombinant human IL-33 (OriGene Technologies, TP760633) (1 μg) was mixed with Esp (1 μg) in 20 μl PBS supplemented with EDTA (5mM). The mixtures were incubated for 30 minutes at 37°C. The reaction products were loaded on SDS-PAGE and blotted onto PVDF membranes for western blotting, as explained in section 2.2.

For IL-33 cleavage site determination, 10 μg of full-length IL-33 was mixed with 10 μg Esp in 50 μl PBS containing 5 mM EDTA. The reaction was incubated for 30 minutes at 37°C. This was followed by mass spectrometry, as explained above.

### 2.6 ELISA: measurement of IgE, IgG1, and IgG4 antibody binding

IgE, IgG1, and IgG4 levels were measured using an indirect ELISA assay previously described (34). In short, ninety-six-well plates were coated with 100 μL of either ECPs or recombinant *S. epidermidis* antigens (Esp or GehD) at a concentration of 5 μg/mL in coating buffer (Candor Bioscience,121125) at 4°C overnight or 1 hour at 37°C.

The plates were washed three times with phosphate-buffered saline (PBS)-Tween buffer (0.1% [vol/vol] Tween 20), and free binding sites were saturated by addition of 150 μL per well of blocking buffer (10% FCS in PBS) for 1 hour at room temperature on a plate shaker at 100 rpm. After that, 50 μL of human serum samples diluted in blocking buffer, 1:50 for IgG1 and IgG4 and 1:5 for IgE, were added to each well and incubated for 1 hour at room temperature and 100 rpm. Detection of bound IgG1 or IgG4 was achieved by incubation with HRP-conjugated secondary antibody, mouse anti-human IgG1 (Thermofisher, A-10648), or -IgG4 (Thermofisher, A-10654) at a concentration of 1 μg/mL in blocking buffer for 1 hour at room temperature and 100 rpm. To measure the IgE levels, a biotin-conjugated secondary antibody (mouse anti-human IgE, 10 μg/mL; antibodies-online, ABIN135676) was used in combination with peroxidase-conjugated streptavidin (3 μg/mL; Dianova, 016-030-084) to detect antibody binding. The plates were washed, and the reaction was developed by adding 50 μl of the activated substrate solution (TMB substrate, BD Biosciences, 555214) and incubated in the dark for 10 min. The reaction was stopped by adding 20 μl of 2 N H_2_SO_4_. Absorbance was measured at 450 nm in an ELISA microtiter reader (Infinite M200 Pro plate reader, Tecan Austria GmbH). Single OD measurements were performed, and the blank value in the absence of the serum was subtracted from the measured sample values.

### 2.7 Human cells

#### 2.7.1 T cell stimulation assay

Whole blood was drawn from healthy individuals and AD patients. Blood was anticoagulated with EDTA, and PBMCs were isolated from 25 mL of blood using Ficoll density gradient centrifugation. CD14+ monocytes were isolated from the PBMCs by positive selection using CD14 MicroBeads (Miltenyi Biotec, 30-050-201) and irradiated with 30 Gy. Untouched T cells were isolated from the CD14-negative fraction using Pan T cell isolation kits (Miltenyi Biotec, 130-096-535) according to the manufacturer’s protocols.

Purified T cells were co-incubated at a ratio of 10:1 with irradiated autologous CD14+ feeder cells in RPMI medium (PAN Biotech, P04-17500) supplemented with 5% human serum (heat-inactivated) (PAN Biotech, P30-2401), 100 IU/mL penicillin, 200 µg/mL streptomycin, 4 mM glutamine, 50 µM β-mercaptoethanol, 1.0 mM sodium pyruvate, 0.1 mM non-essential amino acids (Sigma, M7145-100M). Cells (100,000 T cells; 10,000 monocytes) were seeded in each well of a 96-well plate (round-bottom) and stimulated with 10 µg/mL of heat-inactivated Esp or medium only with a total volume of 200 µL per well. On day 5, 100 µl of the medium was replaced by fresh medium supplemented with 20 IU/mL recombinant human IL-2 (Miltenyi Biotec). On day 9, 120 µL of the supernatant was taken and stored at -80°C until cytokine analysis.

The supernatants’ cytokine concentrations were measured using a 13-plex cytometric bead array (LEGENDplex Human Inflammation Panel, BioLegend 740721) according to the manufacturer’s protocols. Fluorescence signals were captured with an LSR II Flow Cytometer (BD Bioscience), and data were analyzed with the corresponding LEGENDplex software.

#### 2.7.2 Monocyte and monocyte-derived dendritic cells, stimulation with IL-33

Monocytes were isolated from PBMCs by positive selection using CD14 MicroBeads as described above. The monocytes were either directly stimulated with Esp-digested IL-33 or differentiated into monocyte-derived dendritic cells (moDCs) and then stimulated with Esp-digested IL-33.

Monocytes were seeded into round-bottom 96 well-plates (50 000 cells/well in 100 µL medium) in RPMI 5% human serum (heat-inactivated) and stimulated for 24 hours with 100 ng/mL of either IL-33 alone, IL-33 digested with Esp, or Esp alone. After that, the culture supernatants were collected and kept at -80°C until IL-6 measurement.

Human moDCs were generated over 5 days in Opti-MEM medium (ThermoFischer Scientific, 31985070) supplemented with 10% heat-inactivated FCS, 50 ng/mL human IL-4 (Peprotech, 200-04), and 50 ng/mL human GM-CSF (Peprotech, 300-03). On day 0, monocytes were seeded in flat-bottomed 96 well-plate (200 000 monocytes/well in 200 µL medium). Exchange of medium was done on day 2, 150 µL of the culture medium was substituted with fresh Opti-MEM medium (supplemented with IL-4 and GM-CSF). On day 5, the medium was exchanged, and fresh Opti-MEM medium (without supplements) was added to the wells. The moDCs were stimulated as described above for 24 hours; the culture supernatant was collected for IL-6 measurement.

IL-6 was measured using an ELISA Max Deluxe Set (BioLegend, 430504) according to the manufacture’s protocol.

### 2.8 Statistics

Statistical analysis was performed with GraphPad Prism 7.0 software (GraphPad Software, USA). The unpaired nonparametric Mann-Whitney test was used for the comparison of 2 groups, showing non-Gaussian distribution.

## 3 Results

### 3.1 Identification of Esp *of S. epidermidis* as an IgG4-binding protein

**Figure 1:**
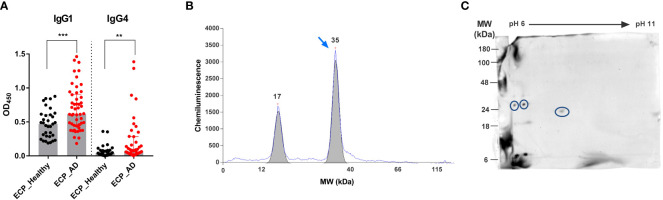
Identification of Esp as an IgG4-binding protein of *S. epidermidis.* **(A)** IgG1 and IgG4 binding to ECPs of *S. epidermidis.* Specific IgG1 and IgG4 antibodies were measured in the sera of 50 AD patients (red) and 30 healthy individuals (black). **(B)** 1D-immunoblot. *S. epidermidis* ECPs were separated by size using a capillary-based automated immune blotting system. Signals from IgG4 binding to the ECPs were obtained. The blue arrow shows a strong signal from IgG4 binding to a protein with a molecular mass of around 35 kDa. **(C)** *S. epidermidis* ECPs were separated by 2D-gel electrophoresis and blotted onto a membrane. The blot was incubated with pooled sera from AD patients and decorated with anti-human IgG4 HRP-conjugated antibodies. The IgG4-binding spots were cut out, and the proteins were identified by LC-MS/MS. Circled spots represent Esp. AD, atopic dermatitis; ECPs, extracellular proteins of *S. epidermidis*; MW, molecular weight; **P≤ 0.01, ***P≤ 0.001, Mann-Whitney U-test.

To study the immune memory of *S. epidermidis* in healthy adults and AD patients, we first compared serum IgG1 and IgG4 binding to the bacterial extracellular proteins (ECPs) using an ELISA. IgG4 served as a surrogate of IgE because both immunoglobulin classes are generated under similar microenvironmental conditions (34, 46). The serum concentrations of *S. epidermidis*-specific IgG1 and IgG4 were much higher in AD patients than in the controls (**Figure 1A**). This indicates extensive exposure of the immune system to *S. epidermidis* and memory formation in AD. Using serum samples from AD patients, automated 1D immunoblots revealed a peak of IgG4 binding at the molecular mass of around 35 kDa (**Figure 1B**). We observed this signal in most individuals.

To identify the responsible bacterial protein, we analyzed IgG4 binding to the ECPs of *S. epidermidis* using 2D immunoblotting followed by mass spectrometry. This revealed Esp as the dominant IgG4-binding protein (**Figure 1C**).

### 3.2 IgG1, IgG4, and IgE binding to Esp in healthy donors and AD patients

To measure specific serum antibodies and to confirm that Esp was correctly identified as an IgG4-binding protein, we expressed the enzyme using recombinant technology, and used it as antigen in an ELISA. Recombinant triacylglycerol lipase (GehD) of *S. epidermidis* was included as a control antigen.

**Figure 2:**
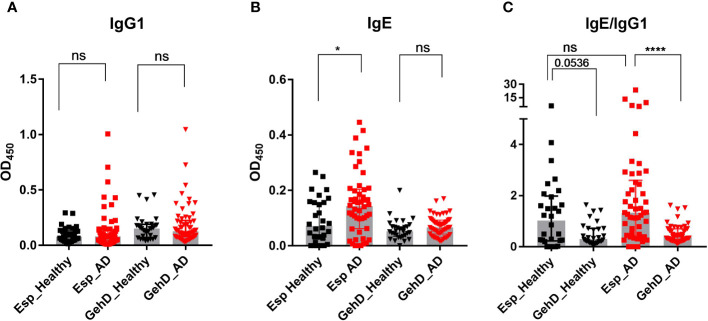
Specific antibody measurement. Specific IgG1 and IgE antibodies against Esp and GehD were measured in the sera of 50 AD patients (red) and 30 healthy individuals (black). **(A)** IgG1 binding to Esp and GehD of S*. epidermidis.* **(B)** Serum IgE specific for Esp and GehD. **(C)** The IgE/IgG1 was calculated using the data presented in **(A, B)**. Medians are shown. AD, atopic dermatitis; Esp, extracellular serine protease; GehD, triacylglycerol lipase; OD, optical density; ns: non-significant, P> 0.05, *P≤ 0.05, ****P≤ 0.0001. Mann-Whitney U-test.

IgG1 binding to Esp and GehD was detectable in most individuals (**Figure 2A**). However, IgG4 binding differed substantially between Esp and GehD: There was strong binding to Esp – with pronounced interindividual differences – but binding to GehD was weak or absent. This was true for both AD patients and healthy controls (**Supplementary Figure S1A**). This difference is highlighted by the IgG4/IgG1 ratios of antibody binding, which were much higher in the case of Esp than GehD (**Supplementary Figure S1B**).

We then asked whether AD patients would develop an allergic response to Esp. Since the hallmark of allergy is the production of specific IgE antibodies, we developed an ELISA to measure specific serum IgE against Esp and GehD. IgE binding to Esp was significantly higher in AD patients than in healthy individuals. In contrast, the level of IgE binding to GehD was low and did not differ between the controls and the AD patients (**Figure 2B**). To compare the IgE binding to Esp and GehD, we calculated the IgE/IgG1 ratios. These corroborated the IgE bias and, hence, the type 2 immune polarizing potential of the bacterial protease Esp (**Figure 2C**).

### 3.3 T cell response to Esp in AD patients and controls

**Figure 3:**
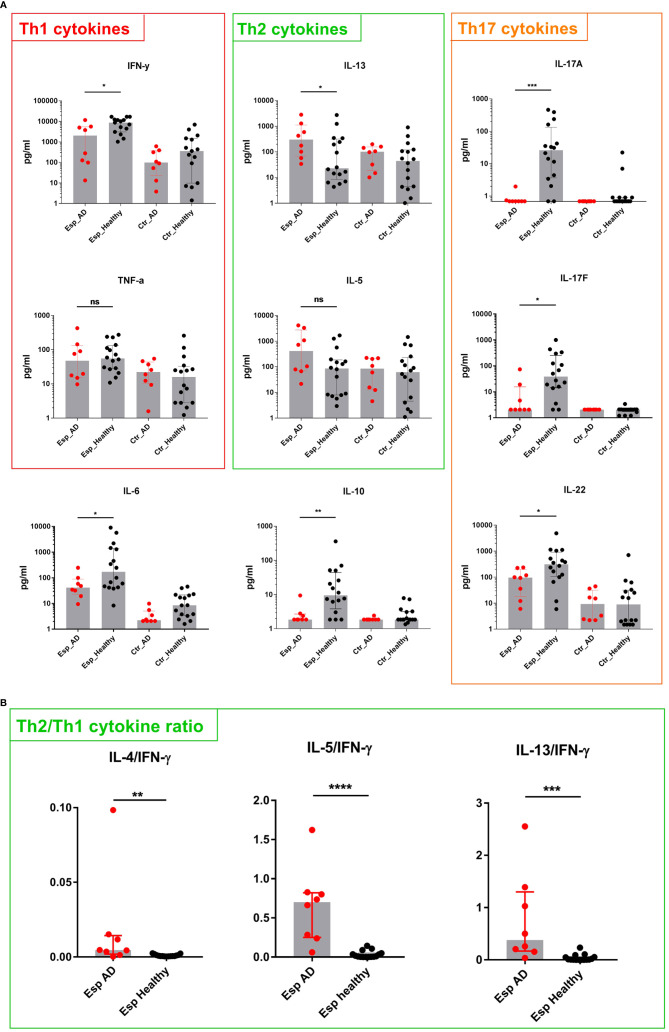
Cytokine secretion by Esp-stimulated T cells. **(A)** T cells and monocytes were isolated from peripheral blood of AD patients (n=8) and healthy individuals (n=16) and stimulated with recombinant Esp. Culture supernatants were harvested on day 9, and cytokine concentrations were measured by cytometric bead array. The T cell response in healthy individuals was dominated by IL-17 and IL-22. T cells from AD patients responded to Esp with a Th2-biased cytokine profile: lack of IL-17 cytokines and reduced IFN-γ, IL-22 as well as IL-10 **(B)** Ratios were obtained by dividing values of Th2 cytokines by those of IFN-γ. Medians are shown. IL, interleukin; AD, atopic dermatitis; Esp, extracellular serine protease; Ctr., unstimulated T cells (medium control). ns: non-significant, P> 0.05, *P≤ 0.05, **P≤ 0.01, ***P≤ 0.001, ****P≤ 0.0001. Mann-Whitney U-test.

To characterize the T cell response to Esp in the 8 AD patients (**Supplementary Table S2**) and the 16 healthy individuals, we measured the secreted cytokines 9 days after stimulation with the recombinant proteins. The cytokine profiles of the T cell response to Esp differed substantially between healthy adults and AD patients. In healthy adults, the Esp-specific response was dominated by Th1/Th17 cytokines (IFN-γ, IL-17A, IL-17F, and IL-22) as well as by IL-6 and IL-10 (**Figure 3A**). Strikingly, the Esp-stimulated T cells from AD patients released almost no IL-17A or IL-17F and only low levels of IL-22 and IL-10. IFN-γ production was also significantly lower in AD than in healthy subjects (**Figure 3A**). In contrast, T cells from AD patients produced more Th2 cytokines than those from healthy adults (significant in the case of IL-13 and by a trend for IL-5). No differences were seen in IL-4 and IL-9 levels (**Supplementary Figure S2**). The ratios of the Th2 cytokines and IFN-γ clearly show the Th2 bias of the Esp-specific T cells in AD as compared to healthy subjects (**Figure 3B**). In summary, the pronounced Th3 polarity of the Esp-specific T cell memory in healthy persons is distorted toward a Th2 profile in AD.

### 3.4 Proteolytic processing of IL-33 by Esp

**Figure 4:**
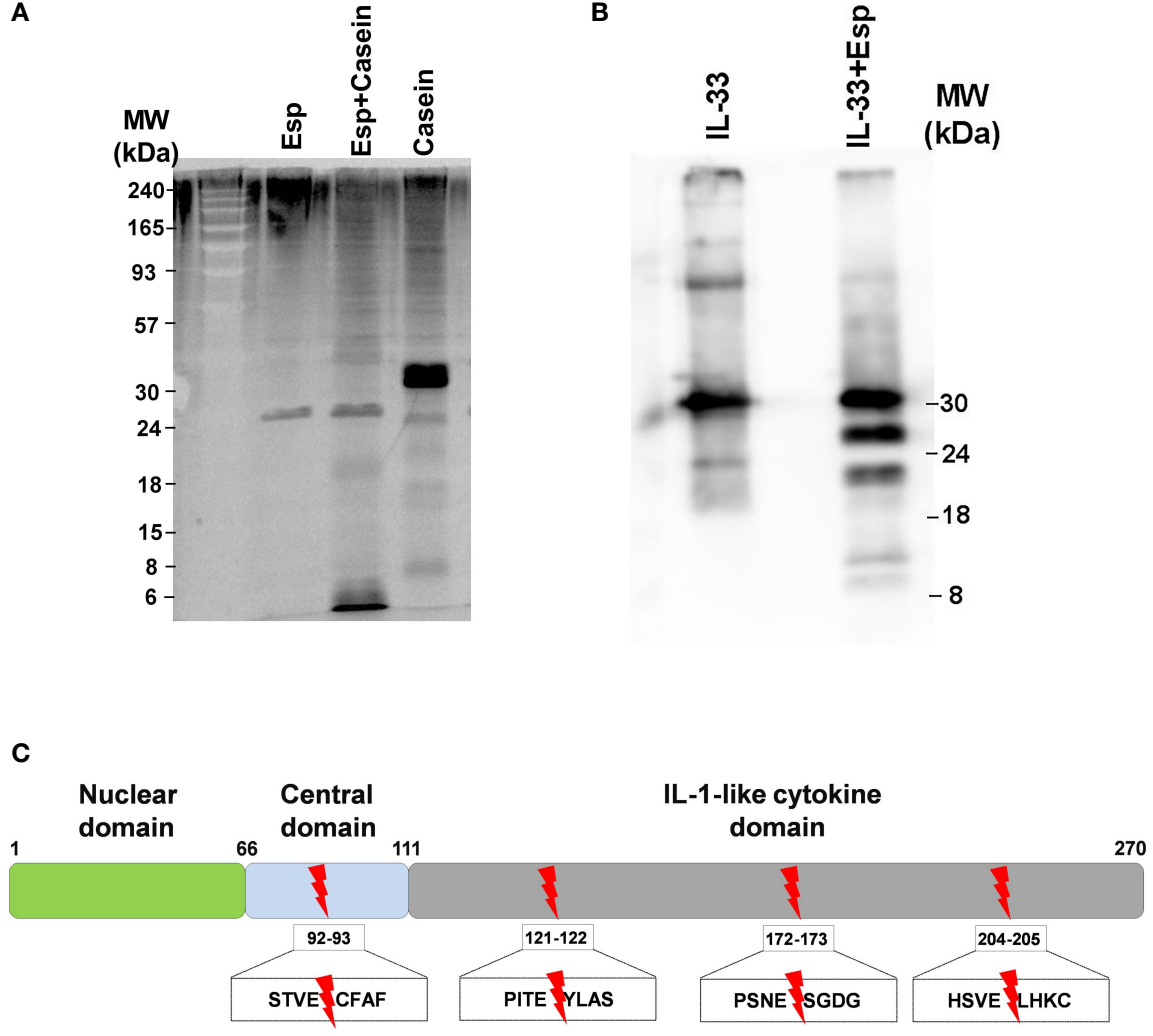
Proteolytic activity of Esp. **(A)** Mature Esp cleaves casein. Casein was incubated for 30 minutes with or without Esp. The reaction products were separated by 12% SDS-PAGE and visualised by chemiluminescence using a protein dye. **(B)** Proteolytic cleavage of IL-33 by Esp. Recombinant full-length IL-33 was digested with Esp for 30 minutes at 37°C. The cleavage pattern was visualised by immunoblotting, using a mouse anti-human IL-33 monoclonal antibody. **(C)** Mass spectrometric identification of the cleavage sites. The red arrows show the location of the Esp cleavage motifs in the IL-33 protein sequence. IL, interleukin; Esp, extracellular serine protease; MW, molecular weight.

**Figure 5:**
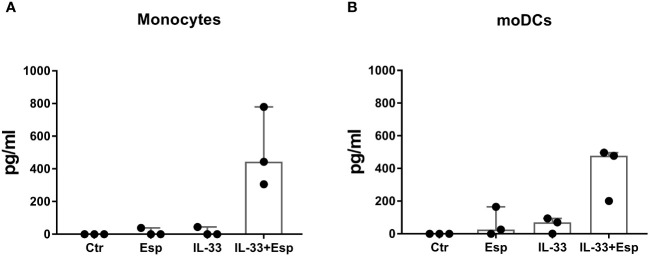
IL-6 response of monocytes and monocyte-derived dendritic cells to Esp-digested IL-33. **(A)** Monocytes were isolated from PBMCs of healthy individuals (n=3) and stimulated with full-length human IL-33, Esp-digested IL-33, Esp alone or medium only (Ctr) for 24 hours (100 ng/mL of each stimulant was used). Secreted IL-6 was measured in the culture supernatant by ELISA. **(B)** Dendritic cells were generated from monocytes (moDCs) from healthy individuals (n=3) and stimulated as described in **(A)**. Both monocytes and moDCs secreted more IL-6 when stimulated with Esp-cleaved IL-33 than in response to the full-length IL-33. moDCs, monocyte-derived dendritic cell; IL-33, interleukin 33; Esp, extracellular serine protease; Ctr, unstimulated medium control.

In search of molecular mechanisms underlying Esp’s type 2 polarizing potential, we reasoned that – similar to other allergenic proteases – Esp might process and activate the alarmin IL-33. We generated enzymatically active Esp by cleaving the recombinant proenzyme with the bacterial protease thermolysin (**Supplementary Figure S3**) and confirmed its proteolytic activity in a casein cleavage assay (**Figure 4A**). The mature enzyme also showed proteolytic activity against recombinant full-length human IL-33. The apparent molecular weight of the generated IL-33 fragments ranged from 10 to 25 kDa (**Figure 4B**). Using mass spectrometry, we identified one cleavage site in the central domain of the cytokine (resulting fragment: IL-33_93-270_) and three cleavage sites in the IL-1-domain (resulting fragments: IL-33_122-270_, IL-33_173-270_, and IL-33_205-270_) (**Figure 4C**, **Supplementary Table S4**). We then tested the activity of the Esp-digested IL-33 using human monocytes and monocyte-derived dendritic cells (moDCs) in cell culture. Measuring IL-6 as the readout, we noticed that monocytes and moDCs produced more IL-6 when stimulated with Esp-processed IL-33 than with the full-length IL-33 (**Figure 5**).

## 4 Discussion

In our search for allergens of *S. epidermidis*, we used IgG4 as a surrogate marker of IgE because IgG4 is present in serum at much higher concentrations than IgE, and both antibody (sub)classes are generated in a similar immunological microenvironment (34). Their production depends on Th2 cells (34, 46). Immunoblotting and mass spectrometry identified Esp as the dominant IgG4-binding protein of *S. epidermidis*. When analyzing the immune response to Esp, we included GehD in our studies as a control antigen because we had previously identified GehD as an immunodominant antigen in *S. epidermidis*-infected patients (data not published). A comparison of human serum antibodies against recombinant Esp and GehD of *S. epidermidis* confirmed the strong IgG4 bias in the humoral response to Esp. Notably, most AD patients and a minority of healthy individuals also had elevated IgE serum levels against Esp, but not against GehD. This corroborates the utility of IgG4 as a surrogate of IgE. It also shows that the strong antibody responses elicited by Esp and GehD of *S. epidermidis* are differently polarized.

Antibody binding to extracellular proteins of *S. epidermidis* and, in particular, Esp-specific IgE serum concentrations were significantly higher in AD patients than in healthy controls, indicating sensitization. This was reflected by the T cell cytokine profile released in response to recombinant Esp. In healthy individuals, this was dominated by type 1/3 cytokines, whereas T cells from AD patients produced almost no IL-17A and IL-17F and only low amounts of IL-22 and IFN-γ. In contrast, the production of IL-5 and IL-13 was higher in AD than in healthy controls. Thus, in AD, the balance between type 1/3 and type 2 inflammation is shifted, and the immune system responds to Esp with IgE and a Th2 cell bias, the hallmarks of the immune reaction to allergens (47, 48).

Some non-AD subjects also had detectable levels of Esp-specific IgE as well as Esp-reactive Th2 cells. This underlines the type 2 immune polarizing potential of Esp and may be explained by the fact that up to 40% of the world’s population have an atopic predisposition (49). In atopic individuals, the immune response against Esp of *S. epidermidis*, which colonizes most humans (50), may be skewed towards a type 2 profile even in the absence of manifest AD or other allergies.

The sensitization to Esp of *S. epidermidis* in AD patients and especially the lack of IL-17 in the patients’ T cell response is remarkable, because usually, the polarity of the adaptive immune response against extracellular bacteria is of type1/3 (51–53). IL-17 is important in controlling bacterial infections (54–57), and shifting the immune response away from the type1/3 profile weakens the anti-bacterial defense. We propose that the type 2-polarising potential of Esp can function as an immune evasion mechanism, paving the way for skin colonization and invasion by *S. epidermidis* and other bacteria (33).

To explain the immune-polarizing potential of Esp, we asked whether the protease can cleave IL-33 and found that the active form of the protease processes the full-length IL-33 into its mature form with increased pro-inflammatory potential. We also observed that Esp cleaves IL-33 in the IL-1-like domain, which is known to inactivate IL-33 (58). Functional assays revealed that the net effect of Esp proteolysis was a strong activation of IL-33. IL-33, a driver of type 2 immunity, is abundant in AD skin (10, 11, 59). Therefore, Esp’s ability to activate IL-33 may contribute to the pathogenesis of AD.

A limitation of this study is the focus on IgG4- rather than IgE binding in our screening for bacterial allergens, because allergens are defined by specific IgE binding. The extremely low concentrations of serum IgE made direct targeting of IgE on immunoblots impossible under our experimental conditions (34). In the future, once candidate allergens have been identified, a basophil activation test would be useful to detect specific IgE, which is mainly bound to the surface of mast cells and basophils, while often very little of the antibodies are circulating (60, 61). We also used an unconventional approach to test the T cell response to the bacterial antigens, measuring cytokines after 9 days of continuous cell culture. In our hands, this increased the sensitivity of the assay, presumably because rare antigen-specific T cells expanded *in vitro*. Although this approach yielded clear results in our pilot study, it should be complemented by more established tests for memory T cells such as analysis of CD154^+^/CD4^+^ T cell-derived cytokines after a shorter *in vitro* recall period or counting Esp-specific CD4^+^ T cells with tetramers in atopic dermatitis patients. Another limitation is the lack of information about *S. epidermidis* colonization in our AD cohort. Therefore, we cannot correlate the presence of *S. epidermidis* on AD skin with disease severity.

Although the *esp* gene is present in 96% of *S. epidermidis* clinical isolates (28) and it is very likely that the protease is released by the bacteria in AD skin, we do not know how the immune system encounters the protease. Esp is too large to penetrate the horny layer of intact skin (Pro-Esp: 31 kDa). However, it is now appreciated that members of the skin microbiome, including *S. epidermidis*, also reside in the dermis, where the secreted protease would be recognized by immune cells (62, 63). Esp could also gain access to the deeper layers of the skin via the lesions caused by EcpA, by virulence factors of *S. aureus*, by inflammation, or by mechanical damage such as scratching (64). It is also possible that sensitization to Esp occurs in the skin or mucous membranes during infection with *S. epidermidis*.

In spite of its immune-polarizing potential, it remains an open question whether Esp is a key pathogenic factor in AD. In future studies, basophil activation testing could be used to confirm that Esp-specific IgE could trigger basophil degranulation in the presence of Esp. This *in vitro* test for the capacity of Esp-specific IgE to trigger degranulation in basophils can be accepted as a surrogate for skin prick testing in research settings and may help to discriminate sensitization to Esp from manifest allergy (60, 61). To date we know that in a mouse model, the ability of *S. epidermidis* to cause severe skin lesions in mice depended on another protease, EcpA, whereas Esp was not sufficient (31). Clearly, the typical type 2 immune reactions are not equally directed against all *S. epidermidis* antigens even in AD, as there was no increased IgE reaction to GehD. We assume that mutual reinforcement of two factors occurs in AD: Esp, an antigen which favors type 2 immunity, and the propensity of an atopic individual’s immune system to Th2 polarization and IgE production.

Our results support the idea that *S. epidermidis*, which is frequently found in AD patients’ skin lesions (29, 65), can aggravate the disease (29, 30). The data presented identify the bacterial protease Esp as a candidate allergen that can cleave and activate the alarmin IL-33. Since Spls, serine proteases of *S. aureus*, also induce a type 2-biased immune reaction, we propose to consider secreted bacterial proteases as candidate allergens.

## Data availability statement

The proteome data presented in this study are deposited via MassIVE to the ProteomeXchange Network and are publicly available. This data can be accessed via https://massive.ucsd.edu/ using the accession number MSV000094880.

## Ethics statement

The studies involving humans were approved by Ethics Committees of Hannover Medical School, Hannover, Germany, and University Medicine Greifswald, Greifswald, Germany (IIIUV 23/06a, BB007/17, MHH7565). The studies were conducted in accordance with the local legislation and institutional requirements. The participants provided their written informed consent to participate in this study.

## Author contributions

GA: Conceptualization, Formal analysis, Investigation, Visualization, Writing – original draft, Writing – review & editing. RP: Investigation, Methodology, Writing – review & editing. LS: Conceptualization, Formal analysis, Investigation, Supervision, Writing – review & editing. MGS: Investigation, Writing – review & editing. JJIG: Investigation, Writing – review & editing. NN: Investigation, Writing – review & editing. DM: Investigation, Writing – review & editing. CS: Methodology, Supervision, Writing – review & editing. UV: Conceptualization, Funding acquisition, Supervision, Writing – review & editing. TW: Conceptualization, Resources, Supervision, Writing – review & editing. BMB: Conceptualization, Funding acquisition, Resources, Supervision, Writing – review & editing. LMR: Conceptualization, Resources, Supervision, Writing – review & editing. LG-G: Conceptualization, Investigation, Supervision, Writing – original draft, Writing – review & editing.
