# Supplementary material for: The extracellular serine protease from Staphylococcus epidermidis elicits a type 2-biased immune response in atopic dermatitis patients
Source: Front Immunol. 2024 Jun 4;15:1352704. doi: 10.3389/fimmu.2024.1352704 (PMC11183529; doi:10.3389/fimmu.2024.1352704)
Supplement: Supplementary file 1 [file DataSheet_1.pdf]

# Supplementary Material

# The extracellular serine protease from *Staphylococcus epidermidis* elicits a type 2-biased immune response in atopic dermatitis patients

**G. Abdurrahman[1], R. Pospich[2], L. Steil[3], M. Gesell Salazar[3], J. J. Izquierdo González[1], N. Normann[1], D. Mrochen[1], C. Scharf[4], U. Völker[3], T. Werfel[2], B. M. Bröker[1*], L. M. Roesner[2], L. Gómez-Gascón[1,5]**

[1]Institute of Immunology, University Medicine Greifswald, Greifswald, Germany

[2]Department of Dermatology and Allergy, Div. of Immunodermatology and Allergy Research, Hannover Medical School, Hannover, Germany

[3]Department of Functional Genomics, University Medicine Greifswald, Greifswald, Germany

[4]Department of Otorhinolaryngology, Head and Neck Surgery, University Medicine Greifswald, Greifswald, Germany

[5]Current address: Department of Animal Health, University of Córdoba; Campus de Excelencia Internacional CeiA3, Córdoba, Spain

## Supplementary Figures and Tables

**Table S1**: Description of AD patients (antibody studies)

| ID | Age (years) | Sex | SCORAD | Severity | total IgE (kU/L) | sx1[a] (kUA/L) |
|---|---|---|---|---|---|---|
| 2019-S-01 | 24 | m | 65.0 | severe | >5000 | 78.2 |
| 2019-S-02 | 56 | m | 73.0 | severe | 23478 | 68.8 |
| 2019-S-03 | 35 | m | 54.0 | severe | >5000 | >100 |
| 2019-S-04 | 35 | f | 69.5 | severe | nd | nd |
| 2019-S-05 | 23 | m | 70.0 | severe | 24 | 2.4 |
| 2019-S-06 | 39 | m | 50.0 | severe | 4402 | >100 |
| 2019-S-07 | 33 | f | 55.8 | severe | 928 | 40.7 |
| 2019-S-08 | 36 | f | 73.0 | severe | 1656 | 49.4 |
| 2019-S-09 | 24 | m | 68.5 | severe | 653 | 10.1 |
| 2019-S-10 | 30 | m | 67.0 | severe | 369 | 29.0 |
| 2019-S-11 | 47 | m | 98.0 | severe | 19661 | 94.7 |
| 2019-S-12 | 22 | f | 58.7 | severe | 642 | 57.0 |
| 2019-S-13 | 26 | f | 51.8 | severe | 63 | 2.0 |
| 2019-S-14 | 31 | m | 77.5 | severe | 4942 | >100 |
| 2019-S-15 | 28 | f | 61.0 | severe | 5756 | 6.2 |
| 2019-S-16 | 20 | f | 55.0 | severe | 205 | 25.9 |
| 2019-S-17 | 22 | m | 50.0 | severe | >5000 | 69.3 |
| 2019-S-18 | 20 | f | 70.0 | severe | 496 | 0.2 |
| 2019-S-19 | 56 | m | 71.0 | severe | 4506 | 75.8 |
| 2019-S-20 | 35 | m | 92.0 | severe | 4499 | 60.9 |
| 2019-S-21 | 57 | m | 94.0 | severe | >5000 | 72.7 |
| 2019-S-22 | 40 | m | 67.0 | severe | 5683 | 81.7 |
| 2019-S-23 | 31 | f | 57.0 | severe | 2812 | 97.2 |
| 2019-S-24 | 31 | m | 72.5 | severe | >5000 | 78.6 |
| 2019-S-25 | 21 | f | 64.2 | severe | 6317 | 61.4 |

**Table S1 continued**: Description of AD patients (antibody studies)

| ID | Age (years) | Sex | SCORAD | Severity | total IgE (kU/L) | sx1[a] (kUA/L) |
|---|---|---|---|---|---|---|
| 2019-L-01 | 41 | m | 21.0 | mild | 2219 | 33.0 |
| 2019-L-02 | 27 | m | 15.0 | mild | 2756 | 35.7 |
| 2019-L-03 | 25 | f | 12.0 | mild | 3478 | >100 |
| 2019-L-04 | 29 | f | 23.0 | mild | 1111 | 64.8 |
| 2019-L-05 | 19 | m | 17.0 | mild | 1185 | 28.8 |
| 2019-L-06 | 23 | m | 24.0 | mild | 16 | 0.4 |
| 2019-L-07 | 40 | f | 9.8 | mild | 83 | 5.6 |
| 2019-L-08 | 33 | f | 15.3 | mild | 1264 | 62.0 |
| 2019-L-09 | 34 | m | 10.0 | mild | 177 | 38.1 |
| 2019-L-10 | 24 | f | 3.8 | mild | 345 | 71.0 |
| 2019-L-11 | 61 | f | 8.1 | mild | 7 | <0.1 |
| 2019-L-12 | 54 | f | 11.0 | mild | 42 | 0.6 |
| 2019-L-13 | 48 | f | 22.0 | mild | 9866 | 73.3 |
| 2019-L-14 | 41 | f | 6.0 | mild | 15 | 4.0 |
| 2019-L-15 | 29 | f | 6.3 | mild | 35 | 0.1 |
| 2019-L-16 | 26 | f | 21.0 | mild | 852 | 88.4 |
| 2019-L-17 | 36 | m | 21.0 | mild | 325 | 53.4 |
| 2019-L-18 | 42 | f | 23.0 | mild | <2,0 | <0.1 |
| 2019-L-19 | nd | m | 11.9 | mild | 288 | 10.7 |
| 2019-L-20 | 49 | f | 22.0 | mild | 221 | 11.4 |
| 2019-L-21 | 21 | f | 23.9 | mild | 665 | nd |
| 2019-L-22 | 31 | m | 10.9 | mild | 179 | 0.3 |
| 2019-L-23 | 57 | m | 16.0 | mild | 35 | <0.1 |
| 2019-L-24 | 22 | f | 20.0 | mild | 219 | 41.4 |
| 2019-L-25 | 35 | f | 18.0 | mild | 26 | 0.3 |

a) Serum IgE specific to common inhalant allergens (birch, grass, rye, mugwort, Cladrosporium, *Dermatophagoides pteronissinus*, cat, dog)

nd: not determined

**Table S2**: Description of AD patients (T cell studies)

| ID | Age (years) | Sex | SCORAD | Severity | total IgE (kU/L) | sx1[a] (kUA/L) |
|---|---|---|---|---|---|---|
| GAN 1 | 33 | m | 28.0 | moderate | 375 | 67.7 |
| GAN 2 | 25 | m | 39.0 | moderate | 9 | 3.3 |
| GAN 3 | 23 | f | 85.7 | severe | 1094 | 64.5 |
| GAN 4 | 40 | m | 50.0 | severe | 4402 | >100 |
| GAN 5 | 20 | m | 50.0 | severe | 11648 | >100 |
| GAN 10 | 27 | m | 26.0 | moderate | 492 | nd |
| GAN 11 | 23 | f | 38.0 | moderate | 772 | 91.1 |
| GAN 14 | 36 | m | 10.9 | mild | 179 | 0.3 |

a) Serum IgE specific to common inhalant allergens (birch, grass, rye, mugwort, Cladrosporium, *Dermatophagoides pteronissinus*, cat, dog)

nd: not determined

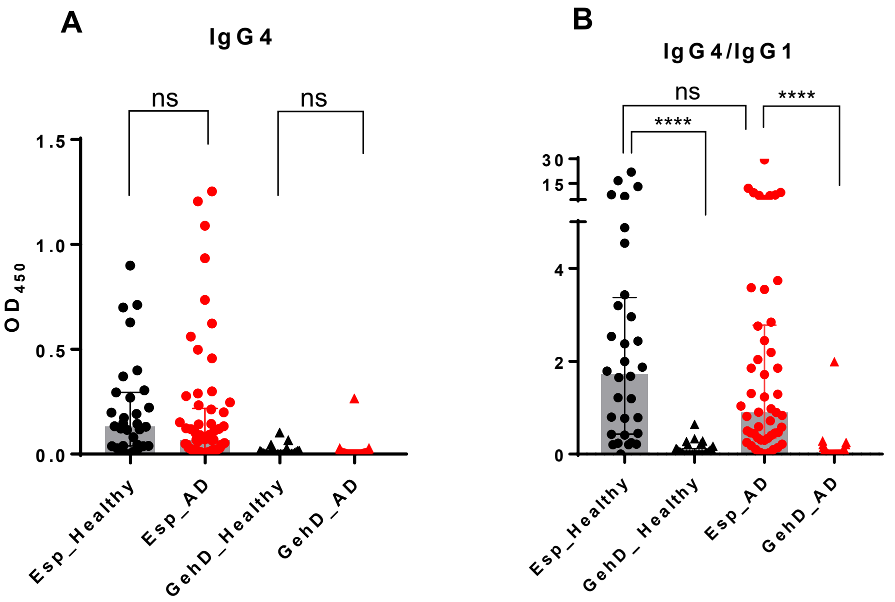


**Figure S1: Specific IgG4 measurements**

Specific IgG4 against Esp and GehD were measured in the sera of 50 AD patients (red) and 30 healthy individuals (black). (**A**) IgG4 binding to Esp and GehD of S. *epidermidis.* (**B**) The ratio of IgG4/IgG1 was calculated using the data presented in Figure 2, A and Figure S1, A. Medians with interquartile ranges are shown. AD: atopic dermatitis, Esp: extracellular serine protease, GehD: triacylglycerol lipase, OD: optical density, ns: P> .05, *P≤ .05, ****P≤ .0001. Mann-Whitney-U test.

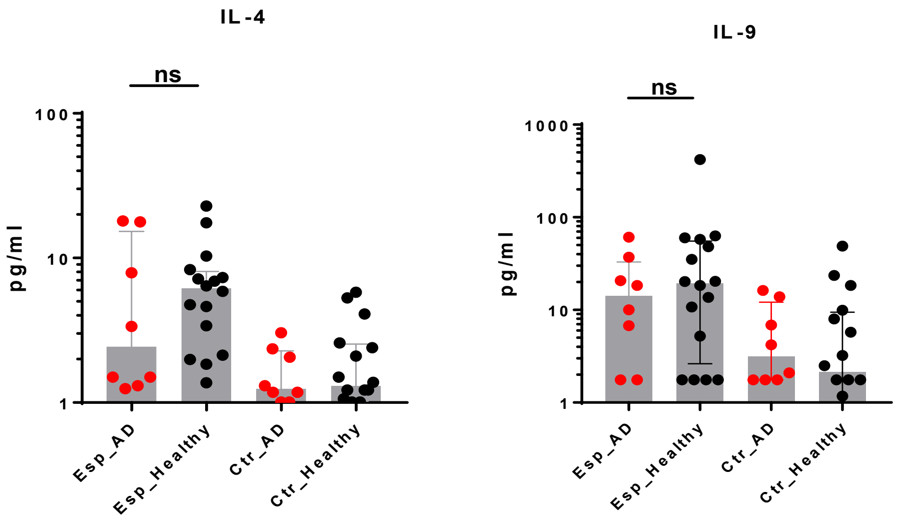


**Figure S2: Cytokine secretion by Esp stimulated T cells**

T cells and monocytes were isolated from whole blood of atopic dermatitis patients (n=8) and healthy individuals (n=16) and stimulated with recombinant Esp. Culture supernatant were harvested on day 9, and cytokine concentrations were measured by cytometric bead array.

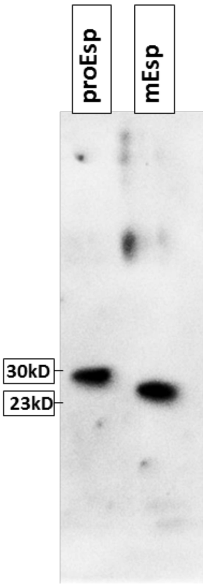


**Figure S3 ProEsp and mature Esp**

The proteolytic maturation of Esp was confirmed by western blotting, targeting the C-terminal His-tag. 0.5ug of each protein was loaded in 12% SDS-PAGE gels and then blotted onto a PVDF membrane. The blot was incubated with mouse anti-His primary antibody and HRP-conjugated goat anti-mouse secondary antibody.

**Table S3**: Nano LC-MS/MS data acquisition parameters.

| | |
|---|---|
| **LC-Parameters** | |
| Instrument | NanoAcquity UPLC (Waters GmbH, Eschborn, Germany) |
| Trap column | NanoAcquity UPLC 2G-V/M trap Symmetry C18 pre-column, 2 cm length, 180 µm ID and 5 µm particle size (Waters GmbH, Eschborn, Germany) |
| Analytical column | NanoAcquity BEH130 C18 column, 10 cm length, 100 µM ID and 1.7 µm particle size (Waters GmbH, Eschborn, Germany) |
| Buffer system | Binary buffer system consisting of buffer A (0.5% DMSO in water with 0.1% acetic acid) and buffer B (5% DMSO in acetonitrile with 0.1% acetic acid) |
| Flow rate | 400 nl/min |
| Gradient – spot identification | 0 min 1% B, 2 min 5% B, 25 min 70% B, 28 min 90% B, 30 min 1% B, 40 min 1% B |
| Gradient – N-terminal enrichment | 0 min 1% B, 2 min 5% B, 30 min 45% B, 32 min 90% B, 34 min 1% B, 40 min 1% B |
| Column oven temperature | 40°C |
| **MS-Parameters** | |
| Instrument | LTQ-Orbitrap Velos mass spectrometer (Thermo Electron Corporation, Germany) |
| Ion Source | Nano-ESI source and installed with a Picotip Emmitter (New Objective, USA). |
| Operation mode | Data-dependent acquisition |
| **Full MS-Parameters** | |
| MS scan resolution | 30,000 |
| AGC target | 1e6 |
| Max. ion injection time for the MS scan | 10ms |
| Scan range | 325 to 1525 m/z |
| Spectra data type | Profile |
| **MS2-Parameters** | |
| MS/MS AGC target | 1e4 |
| Max. ion injection time for the MS/MS scans | 100 ms |
| Selection for MS/MS | 20 most abundant isotope patterns with charge ≥ 2 from the survey scan |
| Isolation width | 2 m/z |
| Dissociation mode | collision-induced dissociation (CID) |
| Normalised collision energy | 35% |
| Dynamic exclusion | 60 s |
| Spectra data type | centroid |
| Charge exclusion | Unassigned, 1, 4 and above |

**Table S4**: Identified peptides of Esp-cleaved IL-33

| Peptide # | Annotated Sequence | Modifications | Position | IL33_ESP/ IL33 (median) | Adj p-value IL33_ESP/ IL33 | Abundance IL33 | Abundance IL33_ESP |
|---|---|---|---|---|---|---|---|
| 1 | CFAFGISGVQKY | 1xCarbamidomethyl [C1] | 93-104 | 20.3 | 0.00636 | 12.8 | 258.8 |
| 2 | YLASLSTYNDQSITF | | 122-136 | 22.9 | 0.00413 | 13.3 | 305.5 |
| 3 | SGDGVDGKML | | 173-182 | 49.6 | 0.00018 | 3.1 | 212.7 |
| 3.1 | SGDGVDGKML | 1xOxidation [M9] | 173-182 | 46.0 | 0.00024 | 8 | 366.7 |
| 3.2 | SGDGVDGKMLMVTL | 2xOxidation [M9; M11] | 173-186 | 11.4 | 0.04392 | 16.2 | 337.2 |
| 4 | LHKCEKPLPDQAFF | 1xCarbamidomethyl [C4] | 205-218 | 71.2 | 0.00003 | 5.2 | 366.7 |
